# Dysregulation of the 3β-hydroxysteroid dehydrogenase type 2 enzyme and steroid hormone biosynthesis in chronic kidney disease

**DOI:** 10.3389/fendo.2024.1358124

**Published:** 2024-10-25

**Authors:** Yiyi Zuo, Dongqing Zha, Yue Zhang, Wan Yang, Jie Jiang, Kangning Wang, Runze Zhang, Ziyi Chen, Qing He

**Affiliations:** ^1^ State Key Laboratory of Oral & Maxillofacial Reconstruction and Regeneration, Key Laboratory of Oral Biomedicine Ministry of Education, Hubei Key Laboratory of Stomatology, School & Hospital of Stomatology, Wuhan University, Wuhan, China; ^2^ Division of Nephrology, Zhongnan Hospital of Wuhan University, Wuhan, China

**Keywords:** chronic kidney disease, metabolomics, HSD3B2, steroid hormone biosynthesis, therapeutic target

## Abstract

**Introduction:**

Chronic kidney disease (CKD) presents a critical global health challenge, marked by the progressive decline of renal function. This study explores the role of the 3β-hydroxysteroid dehydrogenase type 2 enzyme (HSD3B2) and the steroid hormone biosynthesis pathway in CKD pathogenesis and progression.

**Methods:**

Using an adenine-induced CKD mouse model, we conducted an untargeted metabolomic analysis of plasma samples to identify key metabolite alterations associated with CKD. Immunohistochemistry, Western blotting, and qPCR analyses were performed to confirm HSD3B2 expression in both human and mouse tissues. Additionally, Nephroseq and Human Protein Atlas data were utilized to assess the correlation between HSD3B2 and kidney function. Functional studies were conducted on HK2 cells with HSD3B2 knockdown to evaluate the impact on cell proliferation and apoptosis.

**Results:**

Metabolic characteristics revealed significant shifts in CKD, with 61 metabolites increased and 65 metabolites decreased, highlighting the disruption in steroid hormone biosynthesis pathways influenced by HSD3B2. A detailed examination of seven key metabolites underscored the enzyme's central role. HSD3B2 exhibited a strong correlation with kidney function, supported by data from Nephroseq and the Human Protein Atlas. Immunohistochemistry, Western blotting, and qPCR analyses confirmed a drastic reduction in HSD3B2 expression in CKD-affected kidneys. Suppressed proliferation and increased apoptosis rates in HSD3B2 knocked down HK2 cells further demonstrated the enzyme's significance in regulating renal pathophysiology.

**Discussion:**

These findings underscore the potential of HSD3B2 as a clinical diagnostic and therapeutic target in CKD. While further studies are warranted to fully elucidate the mechanisms, our results provide valuable insights into the intricate interplay between steroid hormone biosynthesis and CKD. This offers a promising avenue for precision medicine approaches and personalized treatment strategies.

## 1 Introduction

Chronic kidney disease (CKD) stands as a significant global health concern, affecting over 10% of the worldwide population and resulting from a diverse range of diseases that progressively alter renal structure and function (1). With its debilitating consequences and rising prevalence, CKD has become a focal point in healthcare. The International Society of Nephrology (ISN) reported an alarming 41.5% increase in global absolute CKD prevalence from 1990 to 2017, projecting an impending rise in CKD-related deaths to 2.2 to 4.0 million by 2040 (1–3). The overlap between CKD and cardiovascular disease (CVD) further accentuates the complexity of managing this condition and underscores the need for holistic healthcare approaches (4, 5).

CKD is categorized into stages based on estimated glomerular filtration rate (eGFR) and the presence of kidney damage, with severity increasing as eGFR decreases. The terminal stage, CKD stage 5, also known as end-stage renal disease (ESRD), necessitates artificial filtration or kidney transplantation for survival, severely compromising quality of life (6). Notably, IgA nephropathy and diabetic nephropathy stand out as the two leading causes of end-stage renal failure. Presently, there are no available treatments that have shown the capability to impede the advancement of diabetic and IgA nephropathy to end-stage renal failure (7, 8). Consequently, there is an urgent requirement for innovative therapeutic strategies to proficiently manage diabetic nephropathy. The current therapeutic strategy for CKD mainly centers on mitigating kidney damage progression, achieved primarily through risk factor management and symptom alleviation (9–11). Although these approaches are integral, the identification of novel therapeutic targets for CKD and its associated comorbidities remains a pressing need.

To comprehend the intricate mechanisms underlying CKD, animal models have been instrumental. Among these, the adenine-induced CKD mouse model offers an avenue to study the disease’s pathogenesis without the requirement for surgery or genetic manipulation (12). This model involves administering an adenine-enriched diet, mimicking CKD-like structural and functional changes, such as proteinuria, renal atrophy, fibrosis, and oxidative stress (13–15). It represents a relevant platform for investigating CKD progression.

Metabolomics has emerged as a powerful tool to investigate the underlying molecular intricacies of diseases (16–18). In CKD, it has proven invaluable in identifying biomarkers and shedding light on pathological mechanisms (19). This technology facilitates the profiling of low-molecular-weight metabolites, providing insights into cellular functions, pathways, and metabolic dysregulation associated with CKD (20). Advanced analytical techniques have enabled comprehensive assessments of metabolomic changes, such as alterations in amino acids, lipids, and organic acids, associated with oxidative stress, inflammation, and impaired renal function (18, 21, 22). Metabolomics has the potential to unravel novel diagnostic and prognostic markers and advance personalized medicine in CKD.

This study employs the adenine-induced CKD mouse model and metabolomics analysis to investigate the role of the 3β-hydroxysteroid dehydrogenase type 2 enzyme (HSD3B2) and the steroid hormone biosynthesis pathway in CKD. We explore the potential implications of HSD3B2 and steroid hormone metabolism in CKD pathogenesis, progression, and possible therapeutic interventions. This research not only broadens our understanding of CKD mechanisms but also presents novel avenues for precision medicine approaches tailored to individual CKD patients.

## 2 Materials and methods

### 2.1 Experimental animals and induction of chronic kidney disease

Eight-week-old female C57BL/6 mice were obtained from the Hubei Provincial Center for Disease Control and Prevention (Hubei CDC) and were raised in a specific pathogen-free facility. After 3 days of adaption, animals were divided into two groups: the control group (normal diet, n=8) and the CKD group (0.2% adenine diet, n=6). Chronic kidney disease was induced in mice using the well-established adenine diet model (13), which mimics end-stage renal failure. Mice in the control group received a normal adenine-free diet, while those in the CKD group received a customed diet containing 0.2% adenine (Jiangsu Xietong Bioengineering Co., Ltd) for six weeks. At the end of the study, all mice were anesthetized using a combination of Sutex (75 mg/kg) and xylazine hydrochloride (10 mg/kg), administered via intraperitoneal injection. Following anesthesia, the mice were euthanized by decapitation to obtain blood samples. This method ensured that the animals were fully anesthetized before euthanasia, minimizing any potential suffering.

All animal experiments and procedures performed in this study followed ethical guidelines for animal studies and were approved by the Animal Welfare and Ethics Committee of the School and Hospital of Stomatology at Wuhan University (S07922040A).

### 2.2 Clinical samples

Human kidney biopsy samples were obtained from 6 female patients, including 3 individuals with eGFR higher than 90 mL/min/1.73 m^2^ (Control group) and 3 with eGFR under 30 mL/min/1.73 m^2^ (CKD group), at Zhongnan Hospital of Wuhan University. The staging criteria for CKD were determined by multiple pathologists based on clinical presentation and histological assessment. The experimental protocol was approved by the Medical Ethics Committee of Zhongnan Hospital of Wuhan University (protocol number: 2021074), and all participants provided written informed consent. This study was conducted in accordance with the Declaration of Helsinki.

### 2.3 Biochemical assessment for kidney injury

To evaluate kidney function, blood samples were collected before and at 1, 3, 4, and 6 weeks into the adenine diet. Overnight fasting preceded blood collection. At 0, 1, 3, and 4 weeks, blood samples were obtained by tail nicking into heparin tubes. During euthanasia, whole blood was collected by decapitation. Plasma was separated from blood cells by centrifugation (1,000~2,000 rcf, 10 minutes, 4°C). The supernatant was stored at -80°C until analysis. Serum creatinine (700460, Cayman Chemical, MI, USA), blood urea nitrogen (BUN; 0580-250, Stanbio Laboratory, Boerne, TX, USA), and phosphate concentrations (ab65622, ABclonal, Wuhan, China) were measured using commercial kits respectively, following the manufacturer’s instructions. Levels of adrenocorticotropic hormone (ACTH), interleukin 6 (IL6), and tumor necrosis factor alpha (TNFα) in plasma were measured using enzyme-linked immunosorbent assay (ELISA) kits according to the manufacturer’s protocol (ACTH, E-EL-M0079, Elabscience; IL6, Proteintech, KE10007; TNFα, Proteintech, KE10002). OD values were measured using a microplate reader (Biotech, USA). OD values from at least three independent experiments were analyzed with GraphPad Prism.

### 2.4 Kidney histology

Kidneys were rapidly harvested and fixed in 4% paraformaldehyde (PFA) at 4°C for 24 hours. After dehydration and paraffin embedding, 5 μm sections were cut for histological analysis. Hematoxylin and eosin (H&E) staining was performed to visualize cell morphology, interstitium, glomeruli, and tubules.

### 2.5 Immunohistochemistry

Kidney and adrenal gland sections underwent deparaffinization and hydration through dimethyl benzene and ethanol. Antigen retrieval was achieved by boiling in 10 mM sodium citrate buffer (pH 6.0) for 10 min in a microwave. An HRP polymer anti-rabbit IHC kit (Maixin, Fuzhou, China) was used according to the manufacturer’s instructions. The primary antibody of HSD3B2 (A1823, ABclonal, Wuhan, China) for mouse samples was applied at a 1:10 concentration. The primary antibody of HSD3B2 (122513, Zen Bio, Chengdu, China) for human samples was applied at a 1:50 concentration. Subsequent staining involved using a diaminobenzidine (DAB) reagent kit (Maixin, Fuzhou, China) after incubation with HRP secondary antibody. Slides were sealed and left to air dry overnight at room temperature. As a negative control, the primary antibody was replaced with PBS alone.

### 2.6 Cell culture

Human renal proximal tubule (HK2) cells were purchased from ATCC and cultured in DMEM/F12 medium (C11330500BT, Gibco, China). The medium included 10% fetal bovine serum (FBS; 10270-106, Gibco, Brazil) and 1% penicillin-streptomycin (SV30010, Cytiva, USA). Cells were maintained in a humidified atmosphere of 5% CO2 and 95% air at 37°C. Fresh growth medium was added every 2~3 days until confluent.

### 2.7 Small Hairpin RNA Plasmid Construction and Lentivirus Infection

The shRNA plasmids targeting human HSD3B2 gene (ENSG00000203859) were constructed according to the protocol of the Broad Institute. We inserted the target sequences of human HSD3B2 (ENST00000369416; 201-exon4) into PLKO.1 vector. The circular PLKO.1 plasmid was digested by AgeI (R3552L, NEB, Ipswich, MA, USA) and EcoRI (R3101L, NEB, Ipswich, MA, USA), and the prepared open vector was then ligated with the annealed oligo pair. The ligated plasmid was amplified by transferring it into DH5α (KTSM101L, AlpalifeBio, Shenzhen, China) for clone selection. Finally, the recombinant plasmids were identified by DNA sequencing.

To perform cell transfection, Lipofectamine™ 2000 (Thermo Fisher Scientific, Waltham, MA, USA) was used according to the following protocol. A 2.4 μg plasmid (containing 1.2 μg target plasmid packaged with the 0.9 μg pSPAX2 and 0.3 μg pMD2.G) was mixed with 150 μL serum-free Opti-MEM medium, and then mixed with an additional 150 μL serum-free Opti-MEM medium containing 6 μL Lipofectamine™ 2000. The mixture was incubated at room temperature for 20 minutes and then added to HEK293T cells in 6-well plates. Cells were incubated with the mixture of transfection reagent and plasmid at 37°C for 10 hours before replacement. 48 hours after replacement, recombinant lentivirus was collected and stored at -80°C.

To generate a stable HSD3B2 knockdown cell line, HK2 cells were infected with the recombinant lentivirus for 8 hours and then cultured for 48 hours before stable selection by incubating in a complete medium (as described above) containing 4 μg/mL puromycin dihydrochloride (ST551, Beyotime Biotechnology, Shanghai, China). The medium was changed every 2 days to maintain selection pressure. The primers for shRNA sequences are listed in **Table 1** and were synthesized by Wuhan AuGCT DNA-SYN Biotechnology Co., Ltd (Wuhan, China).

**Table 1:** Primers used for quantitative real-time PCR and shRNA.

Name of genes or shRNAs	Organism	Forward primer	Reverse primer	Product size (bp)
β-actin	Mouse	GATCTGGCACCACACCTTCT	GGGGTGTTGAAGGTCTCAAA	138
HSD3B2	Mouse	GAGATCAGGGTCCTGGACAA	CAATGATGGCAGCAGTATGG	169
β-actin	Human	TGACGTGGACATCCGCAAAG	CTGGAAGGTGGACAGCGAGG	205
HSD3B2	Human	ACAAGGCCTTCAGACCAGAA	ACACAGGCCTCCAACAGTAG	232
HSD3B2-sh1	Human	CCGGATTCCTTTCTGCCAGTA TAAACTCGAGTTTATACTGGC AGAAAGGAATTTTTTG	AATTCAAAAAATTCCTTTCTG CCAGTATAAACTCGAGTTTAT ACTGGCAGAAAGGAAT	
HSD3B2-sh2	Human	CCGGCTTCCTACTCAGCCCAA TTTACTCGAGTAAATTGGGCT GAGTAGGAAGTTTTTG	AATTCAAAAACTTCCTACTCA GCCCAATTTACTCGAGTAAAT TGGGCTGAGTAGGAAG	

### 2.8 Cell viability assay

To assess cell viability, HK2 cells were seeded at 5,000 cells/well in 96-well plates and cultured at 37°C for 24 hours. Subsequently, cells were cultured for varying time points (0, 12, 24, 48, and 72 hours), and viability was determined using the Cell Counting Kit-8 assay (CCK-8; CK04, Dojindo, Kumamoto, Japan) following the manufacturer’s instructions. Briefly, 10 μL of CCK-8 solution was added to each well and incubated for 4 hours at 37°C. Absorbance at 450 nm was measured using a microplate reader (Biotech, USA). OD values from at least six independent experiments were analyzed with GraphPad Prism.

### 2.9 Apoptosis assay

Cells at 90% confluency in complete medium were dissociated using 0.25% trypsin solution (SH40003.01, Cytiva, USA). A cell suspension of 195μl containing 50,000~100,000 cells was prepared with PBS and moved to a centrifuge tube. Staining with Annexin V-fluorescein isothiocyanate (Annexin V-FITC) and propidium iodide-phycoerythrin (PI) was done using an apoptosis detection kit (C1067M, Beyotime Biotechnology, Shanghai, China). After 20 minutes’ incubation in the dark, a flow cytometer (Beckman Coulter, Inc., Brea, CA, USA) was used for the apoptosis assay. Removal of dead cells and debris relied on forward scatter area (FSC-A) vs. side scatter area (SSC-A) analysis, with doublet exclusion using FSC-A vs. forward scatter height (FSC-H). Healthy cells showed negative Annexin V and PI staining, while early apoptotic cells displayed positive Annexin V and negative PI staining. Results indicating the percentage of early apoptotic cells were analyzed using CytExpert version 2.0 software (Beckman Coulter, Inc., Brea, CA, USA). Apoptosis rates from at least three independent experiments were analyzed with GraphPad Prism.

### 2.10 Quantitative real-time PCR analysis

Total RNA was isolated from whole kidneys and HK2 cells using Trizol Reagent (15596026, Ambion, USA) and the HP Total RNA Kit (R6812-02, Omega bio-tek, Norcross, GA, USA), respectively. cDNA synthesis utilized HiScript III qRT Supermix for qPCR (R323, Vazyme, Nanjing, China). Real-time PCR reaction mix contained 2 µL cDNA, 10 µL qPCR SYBR Green Master Mix (11201ES08, Yeasen, Shanghai, China), 0.5µL forward primers (10 µmol/L), and 0.5 µL reverse primers (10 µmol/L), adjusted to 20 µL with ddH2O. RT-PCR was conducted in a two-step procedure: cDNA synthesis (RT, 37°C for 15 min, 85°C for 5 sec), followed by cDNA amplification (95°C for 5 s, 60°C for 30 s) in 39 cycles using a CFX Connect Real-Time PCR Detection System (Bio-Rad, Hercules, CA, USA). Relative gene expression used β-actin as an internal control gene, calculated via the 2-ΔΔCt method. qPT-PCR results are representative of three independent experiments. PCR primer details for each gene are provided in **Table 1**.

### 2.11 Western blot

Kidney tissues were lysed using NP40 lysis buffer (P0013F, Beyotime Biotechnology, Shanghai, China), and protein concentrations were measured using a BCA protein assay kit (23225, Thermo Fisher Scientific, Waltham, MA, USA). For electrophoresis, 30 μg of protein from each sample was separated by 10% sodium dodecyl sulfate–polyacrylamide gel electrophoresis and transferred onto NC membrane (HATF00010, Merck KGaA, Darmstadt, Germany). After blocking for 1 hour at room temperature with 5% nonfat milk in TBST (Tris-buffered saline with 0.05% Tween 20), membranes were incubated overnight at 4°C with primary antibodies: rabbit anti-HSD3B2 (1:500, A1823, ABclonal, Wuhan, China), rabbit anti-vimentin (1:000, PAB30692, Bioswamp, Wuhan, China), rabbit anti-α-SMA (1:1000, PAB30319, Bioswamp, Wuhan, China), and mouse anti-β-actin(1:10000, 66009-1, Proteintech, Chicago, IL, USA). Membranes were washed three times in TBST before incubation with appropriate HRP-conjugated secondary antibodies (goat anti-rabbit, ANT020; goat anti-mouse, ANT020; both from AntGene, Wuhan, China) at a dilution of 1:4000 for 1 hour at room temperature. After three 5-minute washes in TBST, protein bands were visualized with WesternBrightTM ECL solution (Advansta, San Jose, CA, USA) and detected by an Odyssey CLx Imaging System (LI-COR, NE, USA). Protein levels were quantified using ImageJ software (National Institutes of Health, Bethesda, MD). β-actin was used as a loading control. Western blot results are representative of at least three independent experiments.

### 2.12 Untargeted metabolomic profiling

#### 2.12.1 Chemicals and reagents

Analytical grade formic acid (FA) was supplied by Sinopharm Chemical Reagent Co., Ltd. (Shanghai, China). HPLC-grade methanol (MeOH) and acetonitrile (ACN) were supplied by Merck (Darmstadt, Germany). Ultra-pure water used was prepared by a Milli-Q apparatus (Millipore, Bedford, MA).

#### 2.12.2 Sample preparations

Blood samples were collected from the control group (n=8) and the CKD group (n=6) at the end of the 6-week study period. Plasma was separated from blood cells by centrifugation (1,000~2,000 rcf, 10 minutes, 4°C), followed by storage at -80°C until analysis. Plasma samples were thawed at 4°C before use. For each sample, 100 μL plasma was deproteinized by addition of 400 μL MeOH. The mixture was vortexed for 60 s and stored at -20°C for 20 min, and then centrifuged at 13000 rpm for 10 min at 4°C. The supernatant was collected and dried under nitrogen, and then redissolved in 100 μL methanol/water (1/1, v/v) before LC-MS analysis. The quality control (QC) sample was prepared through mixing an equal aliquot (10 μL) from all plasma samples. The same procedure was used for each QC aliquot and individual sample. Throughout the experiment, plasma samples were analyzed randomly, with the QC sample analyzed every 5 samples for the purposes of data filtering, analytical variability evaluation and normalization.

#### 2.12.3 LC-MS analysis

The UHPLC-LTQ-Orbitrap MS system was used for plasma samples analysis, consisting of a Dionex Ultimate 3000 UHPLC system (Thermo Scientific, Sunnyvale, CA, USA) and an LTQ-Orbitrap Elite mass spectrometer (Thermo Scientific, Waltham, MA, USA) with an electrospray ionization source (ESI). LC separation was achieved on a ZORBAX Eclipse Plus C18 Column (50 × 2.1 mm, 1.8 μm, Agilent Technologies) with a flow rate of 0.35 mL/min at 40°C. FA in water (0.1%, v/v, solvent A) and FA in ACN (0.1%, v/v, solvent B) were used as mobile phases. A gradient of 0−1 min at 2% B, 1−9 min from 2% to 98% B, 9−12 min at 98% B, 12−12.1 min from 98% to 2% B, and 12.1−15 min at 2% B was applied. The injection volume was 10 μL.

The MS analysis was performed under both the positive ion mode and negative ion mode with full scan detection of m/z 70-1000 at the resolution of 60000. The ESI parameters were set as follows: heater temperature, 300°C; sheath gas, 35 arb; aux gas, 7 arb or 10 arb for positive or negative mode, respectively; spray voltage, 3500 V; capillary temperature, 350°C. Data-dependent acquisition mode (DDA) was used to acquire the MS2 spectra according to the top 6 intensities with the resolution of 15000. And the MS2 fragment ions were acquired under higher energy dissociation (HCD) with 40% normalized collision energy, 2.0 m/z isolation width, 0.1 ms activation time and 10 s dynamic exclusion duration.

#### 2.12.4 Data Processing and Analysis

Raw data were acquired by Thermo Xcalibur Software (version 2.1, MA, USA). Compound discoverer software (version 3.0) was used for features extraction, retention time correction, peak alignment, and blank subtraction to generate peak table, and QC-based LOESS normalization to generate a comprehensive feature list (23, 24). QC samples were injected in every batch at a regular interval of 5 samples, were used for data filtering. For metabolites detected by two or more platforms, only the values with the lowest relative standard deviation (RSD) in QC samples were retained, and metabolites with RSDs less than 30% in QC samples were selected for further analysis. Before conducting statistical analysis, the data underwent log-transformation to achieve an approximate normal distribution. The detected metabolites were annotated by matching their MS2 spectra against the mzCloud database through Compound discoverer.

For fold change analysis, volcano plot visualization, two-dimensional principal component analysis (PCA), and the identification of CKD-influenced metabolic pathways (Mus musculus), these analyses were conducted using the web-based tool MetaboAnalyst 5.0 (https://www.metaboanalyst.ca/, accessed on 26 January 2023).

### 2.13 Statistical analysis

All data were presented as mean ± standard deviation (SD) from at least three independent experiments. GraphPad Prism software (v. 8.3.1) was used for statistical analyses. Student’s t-test was used for experiments with two groups, while one-way analysis of variance (ANOVA) followed by the Bonferroni *post hoc* test was used for experiments with more than two groups. A P value <0.05 was considered significant.

## 3 Results

### 3.1 Adenine-enriched diet induces chronic renal failure

**Figure 1:**
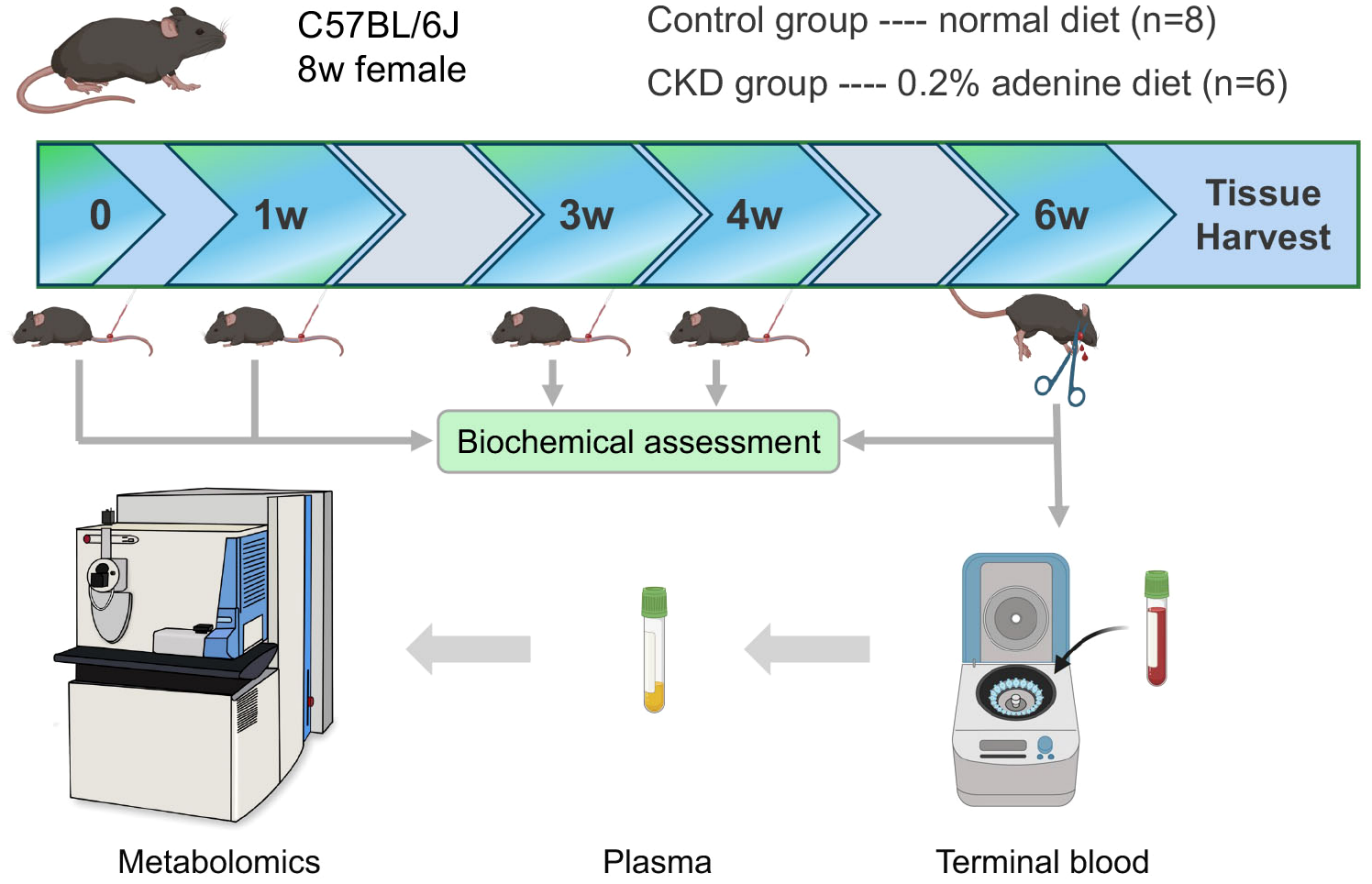
Schematic of the overall experimental workflow performed in this study that involves normal and adenine diet groups of adult female C57BL/6 mice, time points and methods of plasma extraction, and metabolomics analysis.

**Figure 2:**
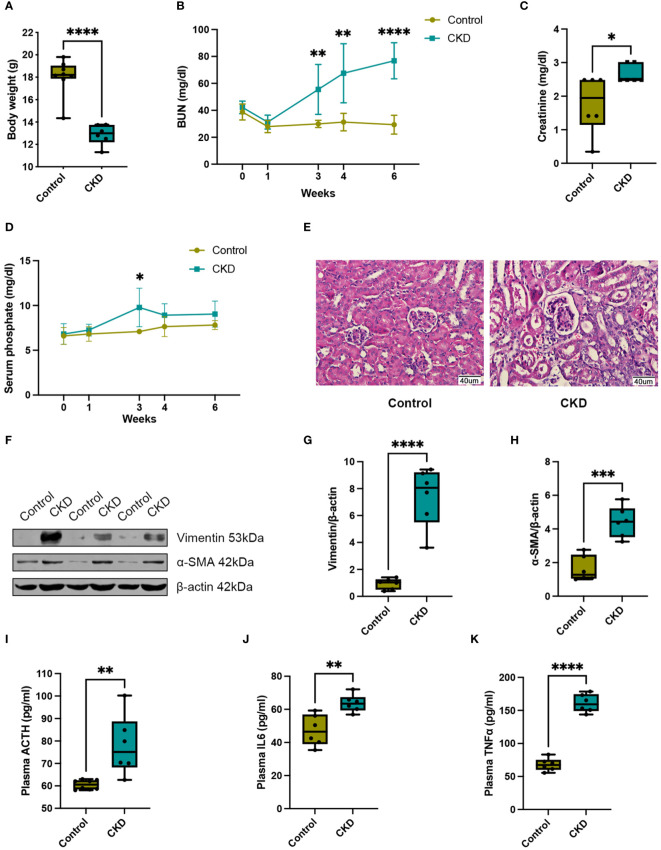
Adenine-enriched diet induced chronic renal failure. **(A)** Box and whisker plots comparing body weight between control and adenine-induced CKD mice at the end of the 6-week feeding period. Control group (n=8), CKD group (n=6). **(B)** Changes of renal function parameters represent by blood urea nitrogen (BUN) concentrations between control and adenine-induced CKD mice. Control group (n=6), CKD group (n=6). **(C)** Box and whisker plots comparing serum creatinine concentrations between control and adenine-induced CKD mice at the end of the 6-week feeding period. Control group (n=6), CKD group (n=6). **(D)** Changes of renal function parameters represent by serum phosphate concentrations between control and adenine-induced CKD mice. Control group (n=5), CKD group (n=5). **(E)** Representative HE staining of kidneys from control or adenine-induced CKD group mice. Scale bar = 40 μm. **(F)** Western blot of vimentin and α-SMA in the kidneys of control or adenine-induced CKD mice. β-actin was used as a loading control. Western blot results are representative of at least three independent experiments. **(G)** Box and whisker plots comparing vimentin and **(H)** α-SMA expression in the kidneys of control and adenine-induced CKD mice. Control (n=6), CKD (n=6). **(I)** Box and whisker plots comparing adrenocorticotropic hormone (ACTH) levels in plasma between control and adenine-induced CKD mice at the end of the 6-week feeding period. Control group (n=8), CKD group (n=6). **(J)** Box and whisker plots comparing interleukin 6 (IL6) levels in plasma between control and adenine-induced CKD mice at the end of the 6-week feeding period. Control group (n=6), CKD group (n=6). **(K)** Box and whisker plots comparing tumor necrosis factor alpha (TNFα) levels in plasma between control and adenine-induced CKD mice at the end of the 6-week feeding period. Control group (n=6), CKD group (n=6). For box and whisker plots, the median expression level is indicated by the central line within each box, while the box represents the interquartile range (IQR). Whiskers extend to the minimum and maximum values within a 1.5 * IQR range. All data are presented as the means ± SEM. Unpaired Student’s t-test was performed to determine the statistical significance, with * p < 0.05, ** p < 0.01, *** p < 0.001, and **** p < 0.0001 versus control group.

In this study, we employed 8-week-old female mice subjected to either a normal or adenine-enriched diet for 6 weeks. Blood samples were collected at intervals of 0, 1, 3, and 4 weeks, with final whole-blood sampling at the 6-week mark (**Figure 1**). At the end of the dietary regimen, mice in the adenine-induced CKD group displayed significant weight loss (**Figure 2A**). Additionally, we assessed renal function markers including blood urea nitrogen (BUN), serum creatinine, and serum phosphate levels. Notably, all markers exhibited marked elevation in the CKD group compared to controls, confirming renal impairment and reduced function in CKD-afflicted mice. BUN levels increased by 1.9-fold (P < 0.01) at the 3rd week, 2.2-fold (P < 0.01) at the 4th week, and 2.6-fold (P < 0.0001) at the 6th week in the CKD group compared to controls (**Figure 2B**). Serum creatinine, a well-established risk factor for morbidity and mortality in CKD patients, significantly rose following the 6-week adenine diet, indicating substantial kidney damage (**Figure 2C**). Moreover, CKD-related renal failure was accompanied by elevated serum phosphate (7.1 ± 0.05 mg/dl in control group vs. 9.8 ± 0.8 mg/dl in AD group, P < 0.05) at the 3rd week after adenine-enriched diet initiation (**Figure 2D**). Hematoxylin-eosin (HE) staining of kidney histological sections showcased distinct features of extensive nephropathy in both renal tubules and glomeruli, confirming CKD-associated histopathological changes (**Figure 2E**). Western blot analysis detected heightened expression of the mesenchymal marker vimentin and α-SMA, indicative of activated fibroblasts, in the kidneys of CKD group mice compared to controls (**Figure 2F**). Statistical evaluation underscored a 4 to 7-fold increase in vimentin and α-SMA in the AD group kidneys (P < 0.001) (**Figures 2G, H**). Together, these findings validate the establishment of the CKD mouse model. Additionally, it has been suggested that perturbations occur in the hypothalamic-pituitary-adrenal (HPA) axis in CKD (25). In line with this, we investigated plasma levels of adrenocorticotropic hormone (ACTH), which were significantly elevated in CKD compared to the controls (p < 0.01), as depicted in **Figure 2I**. We also discovered that plasma cytokine levels, including IL-6 and TNF-α, were significantly increased in CKD mice (**Figures 2J, K**), indicating a heightened inflammatory state.

### 3.2 Plasma metabolome changes in adenine-induced CKD

**Figure 3:**
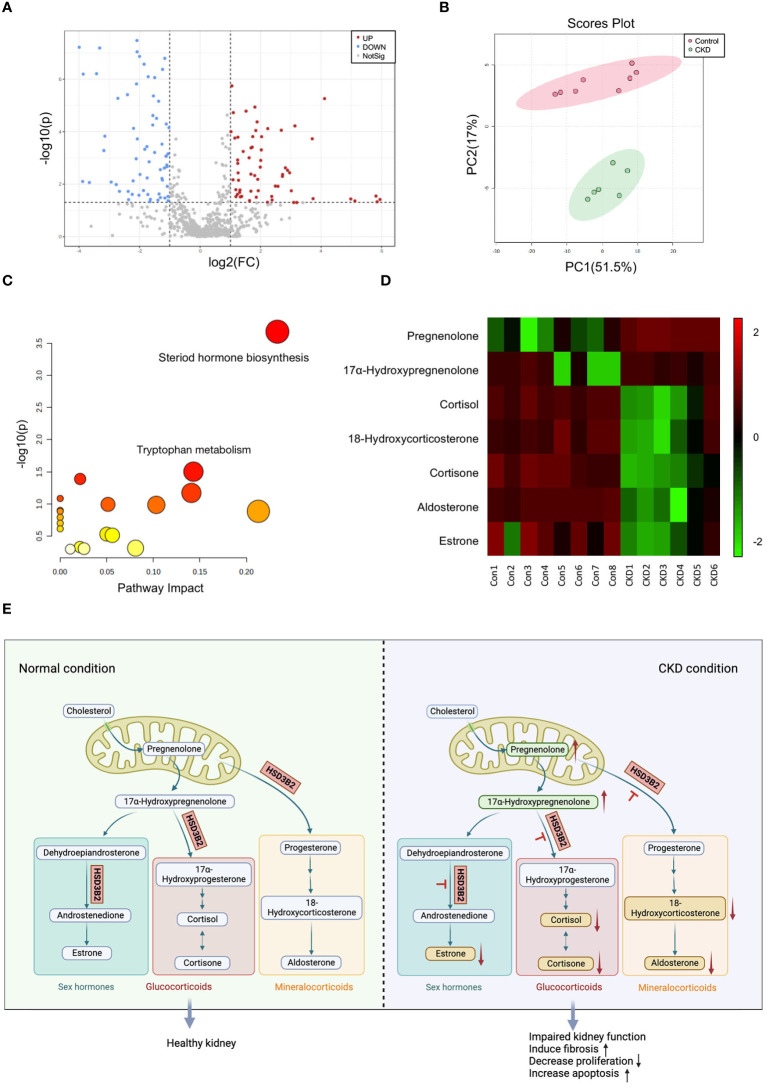
Metabolic analysis of plasma metabolites between control and adenine-induced CKD groups. **(A)** Volcano plots of metabolites that differ between control and adenine-induced CKD groups plasma. Grey dots represent metabolites that were not significantly different between the two groups, while the red and blue dots represent those that are significantly upregulated and downregulated (p < 0.05). **(B)** The 2D PCA analysis comparing the altered metabolites of the plasma samples from control and adenine-induced CKD groups shows two apparent clusters. Red circle and dots represent the control group, while green circle and dots represent the CKD group. **(C)** Analysis of the disordered pathways based on metabolite alterations between control and adenine-induced CKD groups, visualized by a bubble plot. Steroid hormone biosynthesis pathway is mainly identified. The size of the bubble is relative to the impact of each pathway, and the color of the bubble indicates significance, from highest in red to lowest in white; a small p-value and a large pathway impact factor indicate that the pathway is highly influenced. **(D)** Heatmap of the differential metabolites in the steroid hormone biosynthesis pathway measured in the control and adenine-induced CKD groups. Each small square on the X axis depicts individual sample of two groups, while Y axis depicts individual metabolites. The degrees of red or green color indicate higher and lower relative contents of the metabolites. Vertical colored bar is on the right of the heatmap. **(E)** Pathway map illustrating steroid hormone biosynthesis under normal and CKD conditions. Upward arrows indicate upregulated compounds, while downward arrows indicate downregulated compounds.

Untargeted liquid chromatography–mass spectrometry (LC–MS) analysis of plasma samples from 8 control group mice and 6 adenine-induced CKD group mice after 6 weeks of diet revealed 675 identified metabolites. Comparison of relative changes in metabolites unveiled 226 significantly altered compounds. Among these, 94 metabolites were downregulated (log2 FC(Adenine/Control) < -1), while 132 metabolites were upregulated (log2 FC(Adenine/Control) > 1) (**Supplementary Figure 1A**). Notably, the plasma metabolome profiles underwent profound transformations in CKD. A volcano plot confirmed differential clustering, with 126 significantly altered components, comprising 65 downregulated and 61 upregulated metabolites (**Figure 3A**, **Supplementary Table 1**). Two-dimensional principal component analysis (PCA) underscored distinct segregation between control and CKD group samples, reflecting the intricate changes induced by the adenine diet (**Figure 3B**).

### 3.3 Pathway analysis revealed the key pathway and enzyme altered in CKD

Pathway analysis of significantly changed metabolites (p < 0.05) in Metaboanalyst 5.0 illuminated altered functional patterns associated with CKD progression (**Figure 3C**). Notably, steroid hormone biosynthesis emerged as a pivotal pathway of interest, boasting a lower p-value and greater pathway impact factor, enriched with the most differential metabolites. Within this pathway, 7 distinct metabolites underwent significant alterations (**Supplementary Figure 1B**). A heatmap comparing the quantifiable metabolites within this pathway demonstrated marked regulation (**Figure 3D**). Schematic representation of the classic steroid hormone biosynthesis pathway unveiled the elevation of precursors like pregnenolone and 17α-hydroxypregnenolone in CKD plasma (**Figure 3E**). Subsequent metabolic steps yield glucocorticoids, mineralocorticoids, and sex hormones, reliant on hydroxysteroid dehydrogenase (HSD) and cytochrome P450 (CYP) family enzymes. Intriguingly, we observed a reduction in end products such as 18-hydroxycorticosterone, aldosterone, cortisone, cortisol, and estrone. Collectively, elevated precursors and reduced end products suggest an impact on the enzyme HSD3B2, which is pivotal across all branches of this pathway.

### 3.4 HSD3B2 expression was decreased in renal tubular cells of both human and mouse CKD models

**Figure 4:**
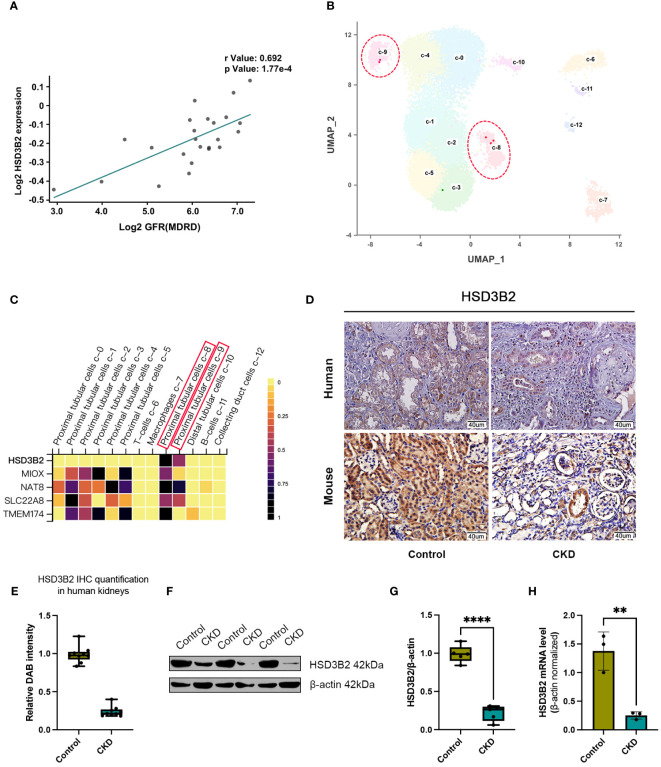
HSD3B2 expression was decreased in renal tubular cells of both human and mouse CKD models. **(A)** Correlation between GFR and renal HSD3B2 expression levels in the Reich IgAN TubInt dataset. HSD3B2 expression level exhibited a positive correlation with GFR in IgA nephropathy samples from CKD patients. **(B)** UMAP clustering of single cell data from the Human Protein Atlas dataset. Cells are clustered and annotated with cluster labels in two dimensions using the dimensionality reduction technique. The cell cluster label in the UMAP correspond to the cell types in the heatmap. **(C)** Heatmap of gene expression levels in different cell clusters of human kidney samples from the Human Protein Atlas dataset. Each small square on the X axis depicts individual cell clusters of the kidney, while Y axis depicts individual genes. Vertical colored bar is on the right of the heatmap. **(D)** Immunohistochemical detection of HSD3B2 in the kidneys of control or CKD group human and mice. Scale bar = 40 μm. **(E)** Box and whisker plots comparing relative DAB intensity of HSD3B2 immunohistochemical staining in control and CKD human kidneys. Control group (n=3), CKD group (n=3). **(F)** Western blot of HSD3B2 in the kidneys of control or adenine-induced CKD group mice. β-actin was used as a loading control. Western blot results are representative of at least three independent experiments. **(G)** Box and whisker plots comparing HSD3B2 expression in the kidneys of control and adenine-induced CKD mice. Control (n=5), CKD (n=5). **(H)** Quantitative real-time PCR analysis of HSD3B2 in the kidneys of control or adenine-induced CKD group mice. Control (n=3), CKD (n=3). β-actin was used as a house keeping gene. qPT-PCR results are representative of three independent experiments. For box and whisker plots, the median expression level is indicated by the central line within each box, while the box represents the interquartile range (IQR). Whiskers extend to the minimum and maximum values within a 1.5 * IQR range. All data are presented as the means ± SEM. Unpaired Student’s t-test was performed to determine the statistical significance, with * p < 0.05, ** p < 0.01, *** p < 0.001, and **** p < 0.0001 versus control group.

Association analysis in the Nephroseq clinical biomarker module revealed correlations between HSD3B2 expression and kidney function indicators, with notable clinical relevance. HSD3B2 mRNA level was closely correlated with GFR in different databases of human samples (**Figure 4A**, **Supplementary Figures 2A, B**). Proteinuria and serum creatinine levels, indicators for renal function, also displayed strong correlation with HSD3B2 mRNA expression (**Supplementary Figures 2C, D**). Expression profiling of western blotting and immunohistochemistry staining collectively demonstrated substantial HSD3B2 presence in renal proximal tubules, a finding validated by single-cell RNA-seq data from the Human Protein Atlas database (**Figures 4B, C**). Immunohistochemistry using human and mouse kidney samples further confirmed this expression pattern (**Figure 4D**). Additionally, a drastic reduction in HSD3B2 expression in CKD-affected kidneys was observed, particularly in atrophied tubules, in both human and mouse tissues (**Figures 4D, E**). Western blot and real-time PCR analyses corroborated these findings, revealing significantly diminished HSD3B2 levels in the adenine-induced CKD group at the 6-week endpoint (**Figures 4F–H**). Collectively, these data underscore the substantial reduction of HSD3B2 in CKD kidneys and imply the critical role of HSD3B2 in maintaining kidney function.

### 3.5 Knockdown of HSD3B2 affects proliferation and apoptosis of HK2 cells

**Figure 5:**
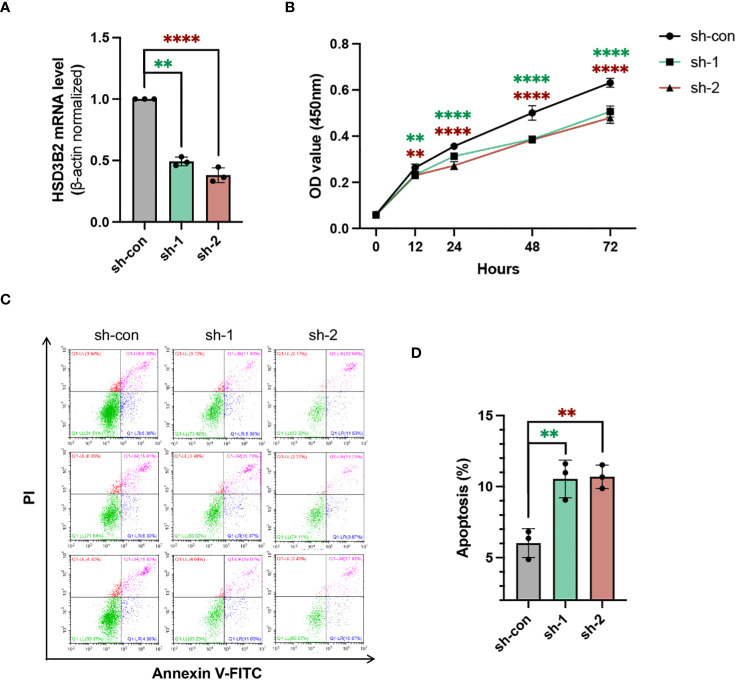
Knockdown of HSD3B2 affects proliferation and apoptosis of HK2 cells. **(A)** Evaluation of the knockdown efficiency of HSD3B2 in HK2 cells following shRNA infection using quantitative real-time PCR analysis (n=3 per group). β-actin was used as a house keeping gene. qPT-PCR results are representative of three independent experiments. **(B)** Cell viability of control and HSD3B2 knockdown groups of HK2 cells was detected by CCK-8 assay (n=6 per group). **(C)** Cell apoptosis of control and HSD3B2 knockdown groups of HK2 cells was detected by flow cytometry analysis of Annexin V and PI staining (n=3 per group). Results of flow cytometry analysis are representative of three independent experiments. **(D)** Quantification of flow cytometry analysis. All data are presented as the means ± SEM. Unpaired Student’s t-test was performed to determine the statistical significance, with * p < 0.05, ** p < 0.01, *** p < 0.001, and **** p < 0.0001 versus control group.

To unravel HSD3B2’s role in CKD renal tubular cells, we employed shRNA targeting HSD3B2 (Human-201-exon4) to knock down HSD3B2 in HK2 cells, a proximal tubular cell line derived from normal kidney. The HSD3B2 targeting shRNAs transduced HK2 cells showed significant knockdown efficiency, exceeding 50% reduction (P < 0.05) by both sh1 and sh2 (**Figure 5A**). Using the CCK-8 assay kit, we assessed HK2 cell viability and observed suppressed cell proliferation upon HSD3B2 knockdown (**Figure 5B**). Apoptosis, a hallmark of CKD-related renal tubular cell pathology, was investigated through Annexin V/PI staining following flow cytometry analysis. The results showed a significant increase in apoptotic cells following HSD3B2 knockdown, compared to the control (**Figures 5C, D**). Collectively, these results highlight HSD3B2’s role in regulating renal tubular cell proliferation and apoptosis within the context of CKD, which contribute to CKD progression.

## 4 Discussion

This study delves into the intricate relationship between the 3β-hydroxysteroid dehydrogenase type 2 enzyme (HSD3B2), the steroid hormone biosynthesis pathway, and chronic kidney disease (CKD). The findings illuminate novel perspectives on CKD pathogenesis and offer potential therapeutic avenues, while acknowledging certain limitations and avenues for future research.

Discovering HSD3B2’s central role in steroid hormone biosynthesis enriches our comprehension of CKD, shifting the focus beyond the traditionally studied hormones like aldosterone and cortisol to a wider spectrum of steroidogenesis impacts on CKD’s advancement (26–29). In some relevant studies on human renal diseases, plasma metabolites were assessed in controls and diabetic subjects with impaired kidney function, revealing a negative correlation between Pregnenolone sulfate and eGFR. Pregnenolone sulfate is one of key precursors in steroid synthesis pathways (30). Our findings of elevated pregnenolone levels in CKD models echo these observations, suggesting a disrupted steroid biosynthesis pathway. Interestingly, early diabetic conditions in rats have been shown to suppress renal steroidogenic activity, pointing to a broader disruption of steroidogenesis in renal diseases (31). However, the specific role of HSD3B2 in CKD progression remained unexplored until now. Our study’s revelation of a marked decrease in HSD3B2 expression in CKD-affected kidneys underscores its potential significance in renal pathology, possibly contributing to key processes like inflammation, fibrosis, and oxidative stress. This opens up new therapeutic possibilities, where modulating HSD3B2 to adjust steroid hormone levels could offer a novel approach to restoring renal balance and slowing CKD progression.

Our investigation into HSD3B2 expression levels revealed that HSD3B2 is also substantially expressed in the kidneys compared to the adrenal glands in wild-type mice (**Supplementary Figures 3A–C**). This finding supports previous reports indicating the kidney’s potential as an extra-glandular steroidogenic organ, with significant transcript levels of HSD3B1 and HSD3B2 detected in kidneys and an observed increase in steroidogenic capacity during development (32–34). Therefore, our results indicate that the decrease in HSD3B2 expression in CKD kidneys may play a significant role in disrupting systemic steroid biosynthesis. Interestingly, our immunohistochemistry staining data indicate that this reduction in kidney HSD3B2 expression does not extend to the adrenal glands (**Supplementary Figure 3D**). This result aligns with previous studies suggesting that late-stage CKD patients may not manifest adrenocortical function failure (35, 36). Thus, the specific decline of HSD3B2 in renal tissue highlights a unique aspect of CKD pathology, suggesting a localized disruption in steroid synthesis that does not affect adrenal gland function.

Understanding the precise mechanisms by which HSD3B2 influences CKD, and the steroid hormone biosynthesis pathway is imperative before translating these findings into therapeutic interventions. Investigating the downstream effects of HSD3B2 dysregulation and its potential interaction with established pathways, such as the renin-angiotensin-aldosterone system (RAAS), will be crucial to assessing its therapeutic potential. Moreover, it’s important to consider the dysregulation of the hypothalamic-pituitary-adrenal (HPA) axis in CKD, which may lead to increased levels of adrenocorticotropic hormone (ACTH). Previous research has highlighted the variability in the function of the HPA axis in CKD patients, particularly regarding ACTH levels (25, 37, 38). While some studies have reported elevated ACTH levels in CKD compared to control groups, others have found no significant differences (39, 40). Interestingly, a discernible trend emerges, suggesting that ACTH elevations are more commonly observed in advanced CKD cohorts, while they may be less evident in the early stages of the disease (29). These findings underscore the complexity of HPA axis dysregulation in CKD and emphasize the need for further investigation into its role in disease progression and management.

In this study, we noted that adenine-induced CKD mice exhibited increased blood viscosity and a shorter coagulation time compared to control mice. This observation is in line with clinical case reports in the literature, which have documented changes in coagulation function and a hypercoagulable state in CKD patients (41). Clinical studies have consistently reported that patients with CKD exhibit altered hemostatic profiles, characterized by an increased tendency for blood clotting (42). These changes include elevated levels of procoagulant factors such as fibrinogen, factor VII (FVII), and factor VIII (FVIII), as well as higher von Willebrand factor (vWF) antigen and activity. These abnormalities contribute to a hypercoagulable state, predisposing CKD patients to thromboembolic events (43). Our findings of increased blood viscosity and faster coagulation in CKD mice induced by an adenine diet mirror these clinical observations, suggesting that this mouse model accurately reflects the hemostatic alterations seen in human CKD.

The identification of HSD3B2’s association with CKD opens new avenues for biomarker discovery. These biomarkers could be pivotal for early disease detection, risk stratification, and predicting treatment responses. Integration of HSD3B2-related biomarkers with other clinical and molecular markers could facilitate precision medicine approaches, tailoring interventions to individual patient needs. Furthermore, deeper insights into HSD3B2’s role in CKD could enable the identification of patient subgroups that may particularly benefit from targeted interventions, enabling personalized treatment plans. For future studies, we propose exploring alternative approaches to modulate HSD3B2 activity in renal tissues. A promising direction could be deploying AAV9 vectors aimed at the kidneys, to boost HSD3B2 expression in renal cells, aiming to restore renal function both *in vivo* and *in vitro*. This novel therapeutic strategy may offer potential benefits with minimal impact on overall steroid metabolism.

Several limitations warrant consideration. The present study only utilized plasma samples for metabolomics analysis. Combining both kidney tissue and plasma samples in the metabolomics analysis could provide a more comprehensive assessment of metabolic pathway changes in CKD, capturing both systemic and renal-specific alterations. Untargeted metabolomic studies involve the simultaneous measurement of metabolites from each sample, aiming to achieve a global and unbiased perspective. However, the vast physiochemical diversity of the metabolome imposes limitations on the number of compounds that can be effectively measured in a single experiment. Factors such as solvent selection, separation strategies, and instrumentation platforms strongly influence which metabolites can be detected (44). Additionally, untargeted metabolomic analysis often relies on comparing experimental spectra with spectral databases for metabolite identification. Nevertheless, these databases may not encompass the entire metabolome, posing challenges in accurately identifying all detected metabolites (45).

All controls and CKD mice utilized in this study were female. The decision to use female mice was based on existing evidence indicating that female mice are more tolerant to adenine supplementation compared to males, which is reflected in decreased mortality rates and delayed occurrence of kidney damage following adenine supplementation (46). Male mice exposed to long-term adenine-enriched diet often exhibit significant reductions in body weight and signs of poor health, making further characterization challenging. It appears that sex hormones may be the cause of greater susceptibility of male kidneys to progressive renal injury in mice (47). Therefore, to ensure the feasibility and reliability of our experimental model, female mice were chosen for this study. While female mice were selected to ensure the study’s viability and consistency, it’s important to note that CKD prevalence globally is higher in women than in men (48, 49). Future studies might benefit from including both male and female subjects to explore gender-related differences in CKD progression and response to interventions.

In conclusion, this study sheds light on the pivotal role of HSD3B2 and the steroid hormone biosynthesis pathway in CKD. The findings expand our understanding of CKD pathogenesis, offering new insights into steroid hormone dysregulation’s potential impact on renal function and pathology. The identification of HSD3B2 as a potential therapeutic target opens exciting possibilities for developing interventions aimed at modulating steroid hormone levels and ameliorating kidney dysfunction. While further research is necessary to elucidate the precise mechanisms and therapeutic potential, these findings provide a foundation for advancing CKD diagnostics and treatment strategies through personalized medicine approaches.

## Data Availability

The metabolomics data presented in the study are deposited in the MetaboLights repository, accession number MTBLS10715 https://www.ebi.ac.uk/metabolights/editor/MTBLS10715/descriptors.
