# Supplementary material for: Dysregulation of the 3β-hydroxysteroid dehydrogenase type 2 enzyme and steroid hormone biosynthesis in chronic kidney disease
Source: Front Endocrinol (Lausanne). 2024 Oct 25;15:1358124. doi: 10.3389/fendo.2024.1358124 (PMC11543464; doi:10.3389/fendo.2024.1358124)
Supplement: Supplementary file 1 [file DataSheet1.pdf]

Supplementary Material

Dysregulation of the 3β-Hydroxysteroid Dehydrogenase Type 2 Enzyme and Steroid Hormone Biosynthesis in Chronic Kidney Disease

Supplementary Figures

A

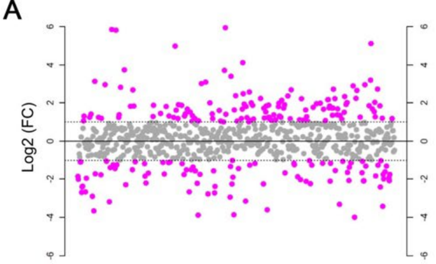

B

Pathway Name	Match Status	p value	=-Log10(p)	FDR	Impact	Matched pathway compounds
Steroid hormone biosynthesis	7/77	0.00020847	3.681	0.017511	0.23319	Pregnenolone; 17alpha-Hydroxypregnenolone; 18-Hydroxycorticosterone; Cortisol; Aldosterone; Cortisone; Estrone;
Tryptophan metabolism	3/41	0.031534	1.5012	1	0.14305	L-Tryptophan; 2-Oxoadipate; Indole-3-acetate;
Pantothenate and CoA biosynthesis	2/19	0.040939	1.3879	1	0.02143	5,6-Dihydrouracil; 3-Methyl-2-oxobutanoic acid;
Lysine degradation	2/25	0.067435	1.1711	1	0.14085	2-Oxoadipate; L-Pipecolate;

Supplementary Figure 1. Metabolomics analysis results of fold change and pathway analysis between the control and adenine-induced CKD groups. (A) Distribution of analyzed metabolites according to the fold change (FC) values. Thresholds were set at FC = 2.0 and FC = −2.0. The result revealed 226 metabolites that differed between control and AD group plasma. The 94 and 132 pink dots depict metabolites that were downregulated or upregulated more than twofold. (B) Summary table of the pathway analysis, where the significantly influenced 3 pathways ($p < 0.05$) are identified, and the top pathway is shown as steroid hormone biosynthesis.

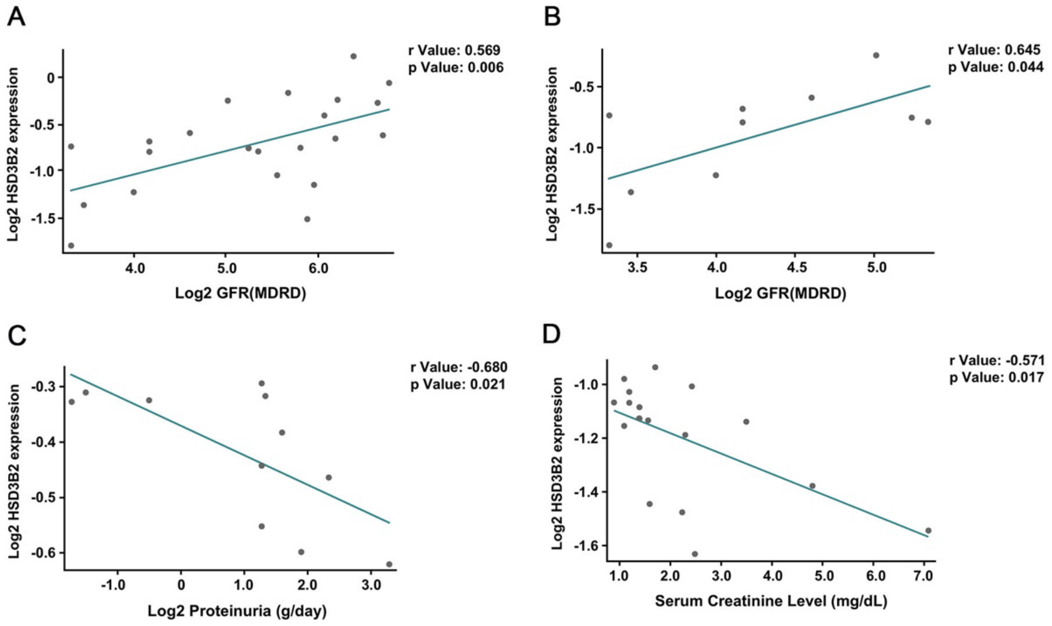

Supplementary Figure 2. Correlation between renal function and HSD3B2 expression level. HSD3B2 expression level was positively correlated with GFR in (A) all measured samples and (B) diabetic nephropathy samples from Woroniecka Diabetes TubInt dataset. (C) Correlation between proteinuria and renal HSD3B2 expression level from Schmid Diabetes TubInt dataset. HSD3B2 expression level was negatively correlated with proteinuria in diabetic nephropathy samples from CKD patients. (D) Correlation between serum creatinine level and renal HSD3B2 expression level from Ju CKD TubInt dataset. HSD3B2 expression level was negatively correlated with serum creatinine level in diabetic nephropathy samples from CKD patients.

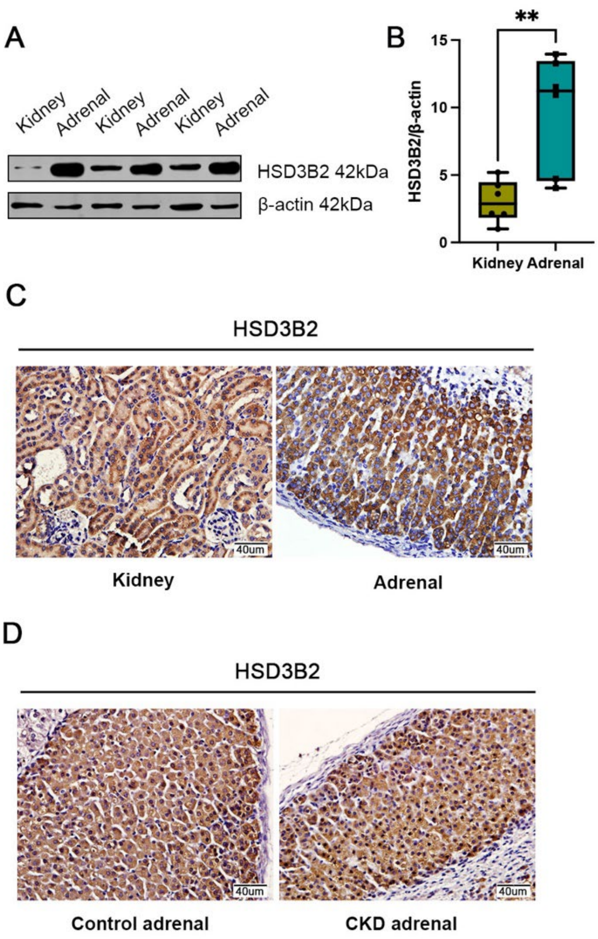

Supplementary Figure 3. Comparison of HSD3B2 expression levels in kidney and adrenal gland of wild-type mouse. (A) Western blot of HSD3B2 in the kidneys and adrenal glands of wild-type mice. β-actin was used as a loading control. Western blot results are representative of at least three independent experiments. (B) Box and whisker plots comparing HSD3B2 expression in the kidneys and adrenal glands of wild-type mice. Kidney (n=6), Adrenal (n=6). The median expression level is indicated by the central line within each box, while the box represents the interquartile range (IQR). Whiskers extend to the minimum and maximum values within a 1.5 * IQR range. (C) Immunohistochemical detection of HSD3B2 in the kidneys and adrenal glands of wild-type mice. Scale bar = 40 μm. (D) Immunohistochemical detection of HSD3B2 in the adrenal glands of control and adenine-induced CKD mice. Scale bar = 40 μm. All data are presented as the means ± SEM. Unpaired Student's t-test was performed to determine the statistical significance, with * $p < 0.05$, ** $p < 0.01$, *** $p < 0.001$, and **** $p < 0.0001$ versus control group.

	log2(FC)	p value
[Similar to: Prostaglandin H1; delta Mass: 25.0199 Da]	-3.9931	6.06E-08
14(S)-HDHA.1	-3.8826	0.007889
(25S)-26-Oxocholest-4-en-3-one	-3.8634	6.40E-07
Ent-7alpha-hydroxykaur-16-en-19-oate	-3.6593	0.0086546
L-Pipecolate.2	-3.4212	6.18E-07
[Similar to: Dothiepin; delta Mass: 77.1625 Da]	-3.3165	6.47E-08
Leucodopachrome	-3.18	0.00052348
Palustradiene-diol.1	-3.1482	0.00014794
Aldosterone	-2.9609	3.30E-04
Dehydroabietadienal	-2.9383	0.0083157
Estrone	-2.8211	0.010335
L-Pipecolate	-2.7158	5.36E-06
Docosahexaenoic acid ethyl ester.1	-2.6637	0.018743
L-Ascorbic acid 2-sulfate	-2.3997	3.91E-06
Linoleate.2	-2.3774	0.018788
11,12-Epoxy-(5Z,8Z,11Z)-icosatrienoic acid	-2.3641	0.038911
[Similar to: 3-(2,5-Dimethyl-1H-pyrrol-1-yl)-2-thiophenecarbohydrazide; delta Mass: 32.0371 Da]	-2.2436	0.024401
Docosahexaenoic acid methyl ester	-2.2141	0.0074776
Cortisone	-2.1281	8.33E-06
[Similar to: PEG n8; delta Mass: -30.9317 Da]	-2.1116	0.0010744
[Similar to: 2-({2-[(4-Fluorophenyl)sulfonyl]ethyl}sulfanyl)-4-(2-thienyl)pyrimidine; delta Mass: 122.0392 Da]	-2.1007	1.52E-06
[Similar to: Cefazolin; delta Mass: 240.0209 Da]	-2.086	3.36E-08
[Similar to: Cefazolin; delta Mass: 218.0388 Da]	-2.0812	9.05E-08
[Similar to: Ursolic acid; delta Mass: 231.2234 Da]	-2.0753	0.0001886
[Similar to: Cytidine 5'-diphosphocholine; delta ass: 59.9659 Da]	-2.0195	0.026431
cis-Aconitic acid.1	-2.0005	1.36E-07
[Similar to: N2-{[(3R,4R,5R)-4,5-dihydroxy-3-{[(4-methylphenyl)carbamoyl]amino}-1-cyclohexen-1-yl]carbonyl}-L-??-glutamine; delta Mass: 157.0449 Da]	-1.9833	0.0042558
[Similar to: Cytidine 5'-diphosphocholine; delta Mass: -103.2557 Da]	-1.8703	0.017414
cis-Aconitic acid	-1.8508	2.69E-07
[Similar to: (7E,13E)-11,12-Dihydroxy-3-isobutyl-4,5,8-trimethyl-3,3a,4,6a,9,10,11,12-octahydro-1H-cycloundeca[d]isoindole-1,15(2H)-dione; delta Mass: 237.1726 Da]	-1.8271	0.00046872
[Similar to: N2-{[(3R,4R,5R)-4,5-dihydroxy-3-{[(4-methylphenyl)carbamoyl]amino}-1-cyclohexen-1-yl]carbonyl}-L-??-glutamine; delta Mass: 174.0714 Da]	-1.7495	0.0057831
[Similar to: 3-Iodo-L-tyrosine; delta Mass: 179.0577 Da]	-1.7277	0.024641
Mesaconate	-1.7158	8.07E-07
(Z)-6'-Hydroxyferulate.1	-1.6836	0.0014064

[Similar to: N-{4-[(2R,3R)-3-(Hydroxymethyl)-4-methyl-5-oxo-2-morpholinyl]phenyl}-3-phenylpropanamide; delta Mass: 225.1151 Da]	-1.6587	0.046308
[Similar to: 2-Methoxy-N-({(2R,4S,5R)-5-[6-(2-methoxyphenyl)-2-methyl-4-pyrimidinyl]-1-azabicyclo[2.2.2]oct-2-yl}methyl)acetamide; delta Mass: 186.1153 Da]	-1.6421	0.037766
Cortisol	-1.6165	0.00066296
2,5-Didehydro-D-gluconate	-1.5611	2.43E-05
[Similar to: alpha-Pinene-2-oxide; delta Mass: 0.0943 Da]	-1.5427	4.36E-06
L-Ascorbate	-1.5015	0.00061785
L-Phenylalanine.2	-1.5003	8.71E-07
18-Hydroxycorticosterone	-1.438	0.0011689
Traumatin	-1.3915	0.040679
[Similar to: 5-Aminovaleric acid; delta Mass: 18.0618 Da]	-1.3697	6.92E-06
Isoquinoline.3	-1.3562	0.023596
5-Acetamidovalerate.2	-1.3114	0.0027897
[Similar to: Withanolide A; delta Mass: 149.0873 Da]	-1.309	0.032321
5-Oxo-L-proline.4	-1.2602	0.0060701
[Similar to: (3beta,5epsilon,9epsilon,18epsilon,19epsilon,20epsilon)-3-(beta-D-Glucopyranosyloxy)urs-12-ene-27,28-dioic acid; delta Mass: 349.2136 Da]	-1.2583	0.00029862
4-Hydroxyphenylacetate	-1.2424	0.0069478
[Similar to: 4-[(1-Cyclopropylethyl)anilino]-4-oxobutanoic acid; delta Mass: 142.0628 Da]	-1.2384	4.24E-07
(1R,4aS)-7-(2-Hydroxypropan-2-yl)-1,4a-dimethyl-9-oxo-3,4,10,10a-tetrahydro-2H-phenanthrene-1-carboxylic acid	-1.1802	0.0024234
[Similar to: 4-[(1-Cyclopropylethyl)anilino]-4-oxobutanoic acid; delta Mass: 159.0894 Da]	-1.1658	1.61E-07
[Similar to: 2-({2-[(4-Fluorophenyl)sulfonyl]ethyl}sulfanyl)-4-(2-thienyl)pyrimidine; delta Mass: 100.1719 Da]	-1.1634	0.033599
[Similar to: L-Kynurenine; delta Mass: -4.0549 Da]	-1.1265	0.00086485
[Similar to: L-Kynurenine; delta Mass: -42.0108 Da]	-1.1213	0.00085048
[Similar to: Galactonic acid; delta Mass: 0.0398 Da]	-1.1178	5.10E-05
8-Hydroxyquinoline	-1.1	0.0018613
Psilocybin.1	-1.0948	0.0021441
Psilocybin	-1.0922	0.00211
4-Hydroxyphenylacetate.1	-1.0836	0.012129
[Similar to: Cycloheximide; delta Mass: 163.0843 Da]	-1.061	0.029259
[Similar to: L-(+)-Citrulline; delta Mass: 17.0263 Da]	-1.0609	0.00069745
Phosphate.1	-1.0567	0.042709
[Similar to: Lasalocid A; delta Mass: 319.2031 Da]	-1.0295	7.04E-05
[Similar to: Cytidine 5'-diphosphocholine; delta Mass: -23.2210 Da]	1.0212	0.00010018
[Similar to: Cytidine 5'-diphosphocholine; delta Mass: -7.2253 Da]	1.0508	1.80E-06
[Similar to: 5-Fluoro-3,5-AB-PFUPPYCA; delta Mass: 0.4134 Da]	1.089	0.0068059

[Similar to: Cytidine 5'-diphosphocholine; delta Mass: -21.2412 Da]	1.0989	1.89E-05
[Similar to: Cytidine 5'-diphosphocholine; delta Mass: -502.5584 Da]	1.1472	0.00017534
Aldosterone.1	1.1816	0.01618
Lapachol	1.184	0.029937
alpha-Linolenate.2	1.2307	0.024654
beta-Cortolone	1.2419	0.0011937
4-Hydroxy-2-oxopentanoate	1.246	0.0016168
[Similar to: L-Glutamine; delta Mass: 0.0732 Da]	1.2504	0.00016181
[Similar to: Citral; delta Mass: 0.1322 Da]	1.2563	0.0037092
[Similar to: 2-(2,5-dimethyl-1H-pyrrol-1-yl)-N'-(2-furylcarbonyl)-4,5-dimethoxybenzohydrazide; delta Mass: 125.3372 Da]	1.2709	0.0071125
Sulfate	1.3085	0.020533
2-Phenyl-1,2,4-triazaspiro[4.4]non-3-ene-3-thiol	1.3184	0.016665
Glycerophospho-N-palmitoyl ethanolamine	1.3313	0.030526
[Similar to: (1R,9R)-11-[(4-Fluorophenyl)sulfonyl]-5-(2-thienyl)-7,11-diazatricyclo[7.3.1.02,7]trideca-2,4-dien-6-one; delta Mass: 240.0124 Da]	1.3759	0.029065
Corticosterone.1	1.4255	0.00056532
4-Indolecarbaldehyde	1.5207	1.65E-05
(+/-)-C75	1.6214	0.00098871
5,6-Dihydrouracil	1.6346	0.042197
Pregn-5-ene-3,20-dione	1.6798	0.018486
Corticosterone	1.6813	0.0041431
[Similar to: trans-4-Hydroxy-L-proline; delta Mass: 17.0111 Da]	1.6865	0.00041431
[Similar to: Indole-3-acrylic acid; delta Mass: 18.0104 Da]	1.7227	0.00038886
[Similar to: 2-({2-[(4-Fluorophenyl)sulfonyl]ethyl}sulfanyl)-4-(2-thienyl)pyrimidine; delta Mass: 122.9093 Da]	1.7411	0.00015377
17-alpha-Hydroxypregnenolone.1	1.8043	0.0046896
Indole.1	1.8117	1.16E-05
[Similar to: 2-({2-[(4-Fluorophenyl)sulfonyl]ethyl}sulfanyl)-4-(2-thienyl)pyrimidine; delta Mass: 100.9273 Da]	1.842	9.04E-05
[Similar to: 2,3-Dinor-11beta-prostaglandin F2alpha; delta Mass: 58.9891 Da]	1.8574	0.031367
N-Methyl-L-glutamate	1.8859	4.23E-05
[Similar to: N-{4-[(2R,3R)-3-(Hydroxymethyl)-4-methyl-5-oxo-2-morpholinyl]phenyl}acetamide; delta Mass: 118.0628 Da]	1.906	0.0065433
[Similar to: Cytidine 5'-diphosphocholine; delta Mass: 80.9359 Da]	1.923	0.017769
[Similar to: Methyl 2-cyano-3-(2-morpholino-5-nitrophenyl)acrylate; delta Mass: 176.0431 Da]	1.9896	0.002242
Indole-3-acrylic acid.2	2.0052	0.0012446
[Similar to: L-(+)-Citrulline; delta Mass: 21.0601 Da]	2.024	0.00049452
[Similar to: 2-(2,5-dimethyl-1H-pyrrol-1-yl)-N'-(2-furylcarbonyl)-4,5-dimethoxybenzohydrazide; delta Mass: 126.0449 Da]	2.0344	0.00015492

17-alpha-Hydroxypregnenolone	2.1942	0.011813
[Similar to: 2,2',5,5'-Tetramethoxy-4,4'-dimethyl-1,1'-biphenyl; delta Mass: 14.0945 Da]	2.2279	0.048926
[Similar to: Methyl 2-cyano-3-(2-morpholino-5-nitrophenyl)acrylate; delta Mass: 201.0510 Da]	2.2383	7.65E-05
[Similar to: (1R,9R)-5-(2-Naphthyl)-11-(propylsulfonyl)-7,11-diazatricyclo[7.3.1.02,7]trideca-2,4-dien-6-one; delta Mass: 172.0244 Da]	2.3686	0.029257
12,13-Epoxy-9,10-trichoene-2-ol	2.3815	0.040219
(25S)-3-Oxocholest-4-en-26-oate	2.5764	0.012057
Indole-3-acetate	2.6858	8.83E-05
[Similar to: N-{4-[(2R,3R)-3-(Hydroxymethyl)-4-isobutyl-5-oxo-2-morpholinyl]phenyl}-2-methoxyacetamide; delta Mass: 100.0886 Da]	2.7223	0.005037
L-Asparagine	2.7272	0.0042633
2-Oxopent-4-enoate.1	2.8207	0.0023817
[Similar to: (+/-)5(6)-DiHET; delta Mass: 165.1978 Da]	2.8935	0.0029467
2-Oxoadipate	2.9616	0.0036695
Levothyroxine	3.0159	0.017869
Haloxyfop	3.0932	0.049671
L-Tryptophan	3.1346	6.02E-05
[Similar to: 6-(4-Chloro-2-cyclohexylphenoxy)pyridin-3-amine; delta Mass: 21.0426 Da]	3.1964	0.049901
Pregnenolone	3.3455	6.48E-04
3,4,15-Triacetoxyscirpenol	3.7087	0.0001862
2-(4-Hydroxyphenyl)-2-oxoacetate	3.7358	0.03566
5,7-Dihydroxy-4-methylcoumarin	4.9825	0.036628
[Similar to: (+/-)5(6)-DiHET; delta Mass: 165.1945 Da]	5.1189	0.043562
Deoxynivalenol	5.816	0.028379
(Z)-6'-Hydroxyferulate	5.853	0.046965
trans-O-Hydroxy-benzylidenepyruvate	5.9477	0.038323

Supplementary Table 1. Differentially expressed metabolites between the control and adenine-induced CKD groups.
